# GABAergic deficits and schizophrenia-like behaviors in a mouse model carrying patient-derived neuroligin-2 R215H mutation

**DOI:** 10.1186/s13041-018-0375-6

**Published:** 2018-06-01

**Authors:** Dong-Yun Jiang, Zheng Wu, Cody Tieu Forsyth, Yi Hu, Siu-Pok Yee, Gong Chen

**Affiliations:** 1 Department of Biology, Huck Institutes of Life Sciences, Pennsylvania State University, University Park, PA 16802 USA; 2 Department of Cell Biology, University of Connecticut Health center, Farmington, CT 06030 USA

**Keywords:** Schizophrenia, GABA, Neuroligin-2, Mouse model, Mutation

## Abstract

Schizophrenia (SCZ) is a severe mental disorder characterized by delusion, hallucination, and cognitive deficits. We have previously identified from schizophrenia patients a loss-of-function mutation Arg^215^→His^215^ (R215H) of *neuroligin 2* (*NLGN2*) gene, which encodes a cell adhesion molecule critical for GABAergic synapse formation and function. Here, we generated a novel transgenic mouse line with neuroligin-2 (NL2) R215H mutation. The single point mutation caused a significant loss of NL2 protein in vivo, reduced GABAergic transmission, and impaired hippocampal activation. Importantly, R215H KI mice displayed anxiety-like behavior, impaired pre-pulse inhibition (PPI), cognition deficits and abnormal stress responses, recapitulating several key aspects of schizophrenia-like behaviors. Our results demonstrate a significant impact of a single point mutation NL2 R215H on brain functions, providing a novel animal model for the study of schizophrenia and neuropsychiatric disorders.

**Electronic supplementary material:**

The online version of this article (10.1186/s13041-018-0375-6) contains supplementary material, which is available to authorized users.

## Introduction

Schizophrenia (SCZ) is a chronic neuropsychiatric disorder caused by both genetic and environmental factors. It is featured by long-standing delusion and hallucination (psychosis), and cognitive deficits [17, 27, 39]. SCZ is a highly heritable disorder [55] with a complex genetic basis. Recent genomic studies identified a number of genetic variants associated with SCZ, including a group of variants resided in the genes encoding synaptic adhesion molecules that promoting synaptic development and function such as *IGSF9B*, and *NLGN4X* [52].

Neuroligins (NLGNs) are a family of synaptic adhesion molecules highly expressed in the brain and are ligands for another group of cell adhesion molecules neurexins (NRXNs) [26]. There are five neuroligin genes (neuroligin-1, − 2, − 3, − 4, and − 5) in humans and four in mice (neuroligin 1–4). Neuroligin-1, − 2, and − 3 are close homologs between human and mice. Neuroligin-1 and neuroligin-2 differentially locate to excitatory and inhibitory synapses and are critical for the excitatory and inhibitory synapse formation and function, respectively [9, 12, 35, 44, 51, 53, 59]. Neuroligin-3 locates at both type of synapses and contributes to both neurotransmission [7, 14, 57]. In recent years, genetic variants of neuroligin-1, neuroligin-3 and neuroligin-4 have been identified in autism patients [28, 43]. Mutations in proteins interacting with neuroligins such as Neurexin1, SHANK and MDGA have also been associated with autism and schizophrenia patients [6, 13, 31, 32]. Genetic mouse models based on these findings recapitulate several aspects of patient symptoms, providing an entry point for the mechanistic study and drug development on psychiatric disorders [2, 10, 14, 15, 29, 46, 50, 54, 57, 66].

We have previously reported several novel mutations of *NLGN2* from schizophrenia patients [56]. Among the NL2 mutants, we found that the R215H mutant protein was retained in the endoplasmic reticulum (ER) and could not be transported to the cell membrane, resulting in a failure to interact with presynaptic neurexin and a loss of function in GABAergic synapse assembly [56]. Based on these studies, we have now generated a transgenic mouse line carrying the same NL2 R215H mutation to test its functional consequence in vivo. We demonstrate that the R215H knock-in (KI) mice show severe GABAergic deficits and display not only anxiety-like behavior seen in global NL2 KO mice [1, 3, 61], but also impaired pre-pulse inhibition, cognitive deficits, and abnormal stress responses which are not reported in global NL2 KO mice. Our results suggest that a single-point mutation R215H of NL2 can result in significant GABAergic deficits and contribute to SCZ-like behaviors. This newly generated NL2 R215H KI mouse may provide a useful animal model for the studies of neuropsychiatric disorders including SCZ.

## Results

### Generation of neuroligin-2 R215H mutant mice

**Fig. 1:**
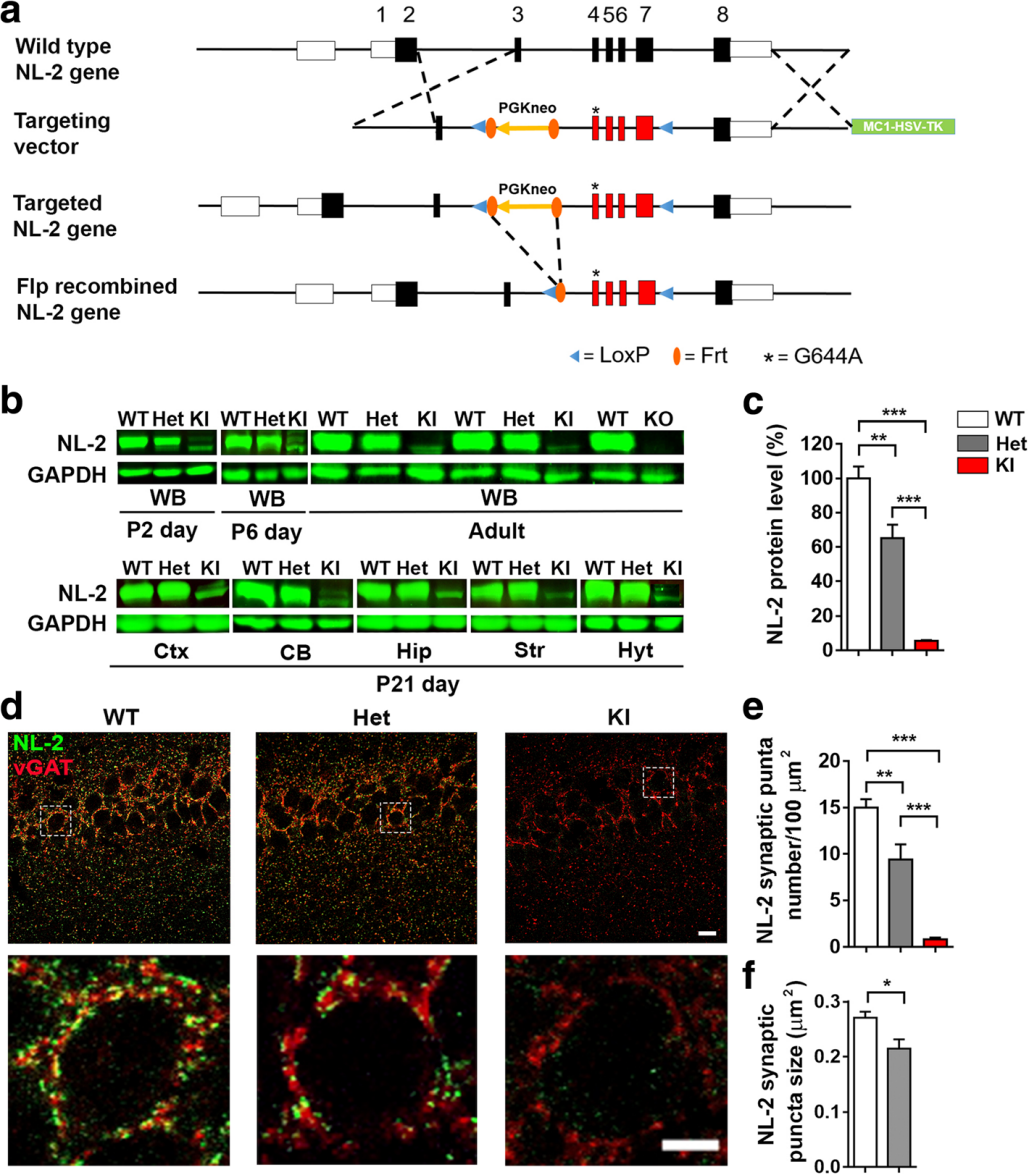
Generation and characterization of NL2 R215H mice. **a** A simplified diagram of NL2 R215H homologous recombination strategy. **b** Representative Western blot of NL2 protein expression at postnatal 2 days, 6 days, 21 days, and adult stage of WT, NL2 R215H Het, and NL2 R215H KI mice. GAPDH was used as internal control. **c** Quantification of NL2 protein expression level in littermates. WT, *n* = 6 mice, Het, *n* = 5 mice, and KI, *n* = 6 mice. One-way ANOVA with post-hoc Tukey multi-comparison test was used for statistical analysis. **d** Representative images of NL2 postsynaptic puncta in WT, NL2 R215H Het and NL2 R215H KI mice. Images were taken at hippocampal CA1 region. Upper row scale bar = 10 μm. Bottom row scale bar = 5 μm. **e**, **f** Quantification of NL2 puncta number and size. Nine brain slices from 3 mice for each genotype were used for analysis. One-way ANOVA with post-hoc Tukey multi-comparison test was used for statistical analysis in (**e**). Student’s *t*-test was used for statistical analysis in (**f**). Data were shown as Mean ± SEM, *P < 0.05, **P < 0.01, ***P < 0.001

Following our original discovery of a loss-of-function mutation R215H of NL2 in SCZ patients [56], we generated the NL2 R215H mutant mice by introducing the same R215H mutation into the exon 4 of *Nlgn2* gene in the mouse genome via homologous recombination (Fig. 1a). NL2 R215H heterozygotes were mated to obtain wild type (WT), heterozygotes (referred here as Het mice), and homozygotes (referred here as KI mice) (Additional file 1: Figure S1a). Sequencing analysis confirmed the R215H mutation in the NL2 KI mice (Additional file 1: Figure S1b). Mice carrying R215H mutation were born at a normal Mendelian rate (Male mice: WT = 26.5%, Het = 52.9%, KI = 20.6%; Female mice: WT = 24.1%, Het = 52.8%, KI = 23.1%). Both R215H Het and KI mice were viable and fertile and did not exhibit premature mortality. During development, we observed a reduction of body weight in R215H KI mice comparing to R215H Het and WT mice in large litters (litter size > 6, Additional file 1: Figure S2a-b), but this phenomenon is not significant in small litters (litter size < 5, Additional file 1: Figure S2c). Body length and tail length is not significantly different between genotypes (Additional file 1: Figure S2d-g). The mouse colony was maintained on a hybrid genetic background to avoid the artificial phenotype contributed by other homozygous genetic variants in a homozygous inbred background.

### Reduction of neuroligin-2 protein level in NL2 R215H KI mice

After obtaining the NL2 R215H Het and KI mice, we first analyzed the NL2 protein expression level in the brain. We found that as early as postnatal 2 days, NL2 already showed substantial expression in the WT mice (Fig. 1b, top row). In NL2 R215H Het mice, the NL2 protein level was about half of the WT mice; whereas in R215H KI mice, the NL2 level was very low comparing to the WT level, but with a clear band of lower molecular weight representing non-glycosylated immature NL2 R215H protein [56, 65]. Such immature band of NL2 R215H protein persisted at adult stage in the KI mice, and was observed in a variety of brain regions (Fig. 1b, and quantified in Fig. 1c). In contrast, NL2 KO mice showed a complete absence of NL2 without any immature band at all (Fig. 1b, top row). Such difference of NL2 protein level between our KI mice and previous KO mice may underlie their functional difference reported later. To test whether NL2 R215H mutation affects the expression of other NL family members, we examined the protein level of NL1 and NL3 in both NL2 R215H Het and KI mice but found no significant changes (Additional file 1: Figure S3).

To investigate the localization of NL2 R215H proteins inside the brain, we performed immunohistochemistry with NL2-specific antibodies and found a significant reduction of NL2 puncta in R215H Het mice and almost absence of NL2 puncta in homozygous R215H KI mice (Fig. 1d-f). In WT mouse brains, NL2 formed numerous postsynaptic puncta on cell soma and dendrites opposing presynaptic vGAT puncta (Fig. 1d-f, puncta density 15.0 ± 0.9 per 100 μm^2^, puncta size = 0.27 ± 0.01 μm^2^). The number and size of NL2 puncta were significantly reduced in the NL2 R215H Het mouse brains (Fig. 1d-f, puncta density, 9.4 ± 1.6 per 100 μm^2^, *p* < 0.01, puncta size, 0.21± 0.02 μm^2^, *p* = 0.02). Interestingly, in the homozygous NL2 R215H KI mouse brains, only faint NL2 signal was observed inside cell soma (Fig. 1d, right columns, Additional file 1: Figure S4) and not colocalized with vGAT, further suggesting that the NL2 R215H proteins could not be transported to the cell membrane [56]. To get a clear understanding of the physiological role of NL2 R215H mutation in vivo, we focused our studies on the homozygous NL2 R215H KI mice in this study.

### Reduced GABAergic synapse density in NL2 R215H KI mice

**Fig. 2:**
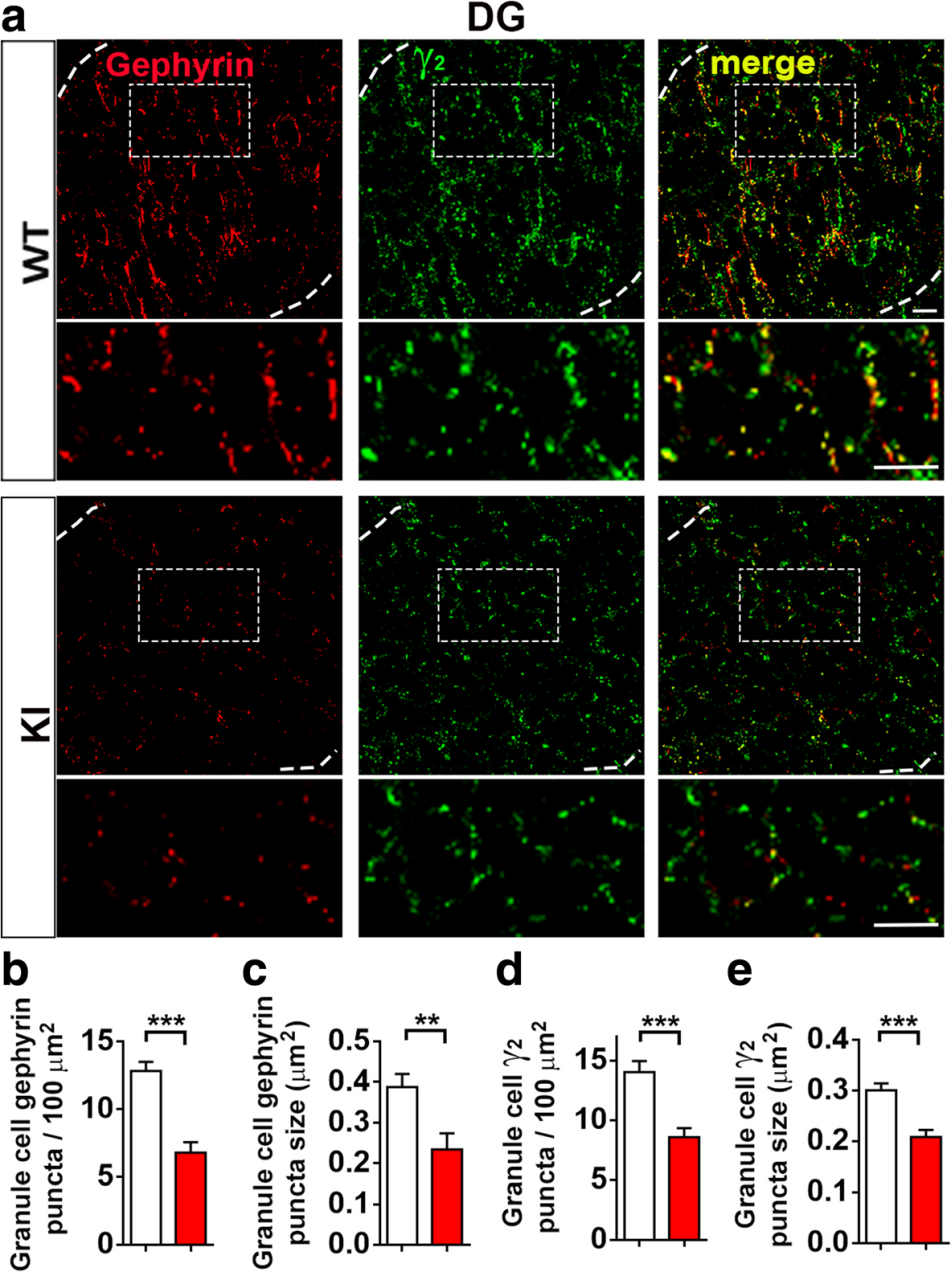
Reduced GABAergic postsynaptic components in the hippocampus of NL2 R215H KI mice. **a** Representative images of gephyrin, GABA_A_ receptor γ2 subunit and merged immunostaining in the granule cell layer of WT and R215H KI mice. **b**, **c** Quantification of gephyrin puncta number and size at granule cell soma region. **d**, **e** Quantification of γ2 puncta number and size at the same region as gephyrin. WT = 9 slices from 3 mice, R215H KI = 12 slices from 3 mice. Scale bar = 10 μm. Student’s *t*-test was used for analysis and data were shown as Mean ± SEM, *P < 0.05, **P < 0.01, ***P < 0.001

**Fig. 3:**
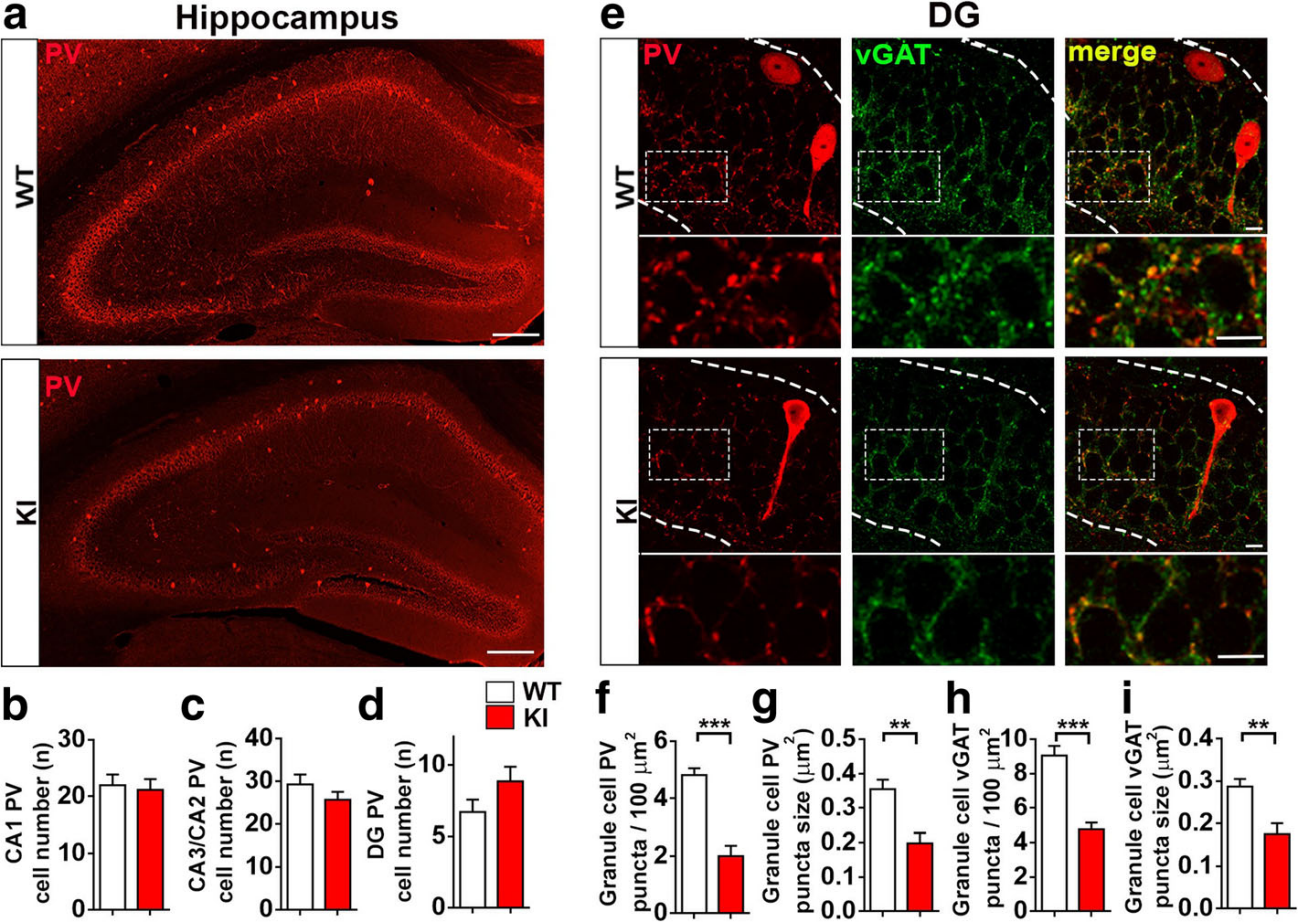
Reduced GABAergic presynaptic components in the hippocampus of NL2 R215H KI mice. **a** Representative images of PV staining at the hippocampus in WT and R215H KI mice. Scale bar = 200 μm. **b**-**d** Quantification of PV-positive neurons at DG, CA2/3, and CA1 region. WT, *n* = 14 slices / 5 mice; KI, *n* = 14 slices / 5 mice. **e** Representative images of PV, vGAT and merged immunostaining in the granule cell layer of WT and R215H KI mice. **f**, **g** Quantification of PV puncta number and size that targeted to the granule cell layer. **h**, **i** Quantification of vGAT puncta number and size that targeted to the same region. WT = 12 slices / 5 mice, R215H KI = 12 slices / 5 mice. Scale bar = 10 μm. Student’s *t* test was used for analysis and data were shown as Mean ± SEM, *P < 0.05, **P < 0.01, ***P < 0.001

NL2 has been reported to form complex with gephyrin and collybistin at postsynaptic sites to recruit GABA_A_ receptors [47]. Consistent with a substantial reduction of NL2 puncta in the KI mice, we detected a remarkable decrease of postsynaptic GABA_A_ receptor γ2 subunit and the scaffold protein gephyrin around cell soma in hippocampal regions (Fig. 2a). Quantitative analysis revealed that both the puncta number and size of postsynaptic γ2 subunit and gephyrin decreased significantly in homozygous R215H KI mice (Fig. 2b-e), consistent with previous findings in NL2 KO mice [1, 19, 30, 47]. In addition to postsynaptic changes, we also examined presynaptic marker vGAT (vesicular GABA transporter) and parvalbumin (PV) positive GABAergic neurons that are reported to be associated with SCZ patients [37]. Immunohistochemistry analysis revealed that the number of PV neurons was not changed in the hippocampal area of the KI mice (Fig. 3a-d). However, both PV and vGAT puncta number and size were significantly reduced in the dentate granule cells (Fig. 3e-i), as well as in the CA1/CA3 pyramidal cells in the KI mice (Additional file 1: Figure S5a-j). Consistently, we observed a reduction of PV and vGAT protein level in the hippocampal tissue of KI mice (Additional file 1: Figure S6a-b). In contrast, the excitatory presynaptic marker vGluT1 was not altered in R215H KI mice (Additional file 1: Figure S6c-e). These results suggest that NL2 R215H mutation impaired both pre- and post-synaptic GABAergic components.

### Impaired GABAergic neurotransmission in NL2 R215H KI mice

**Fig. 4:**
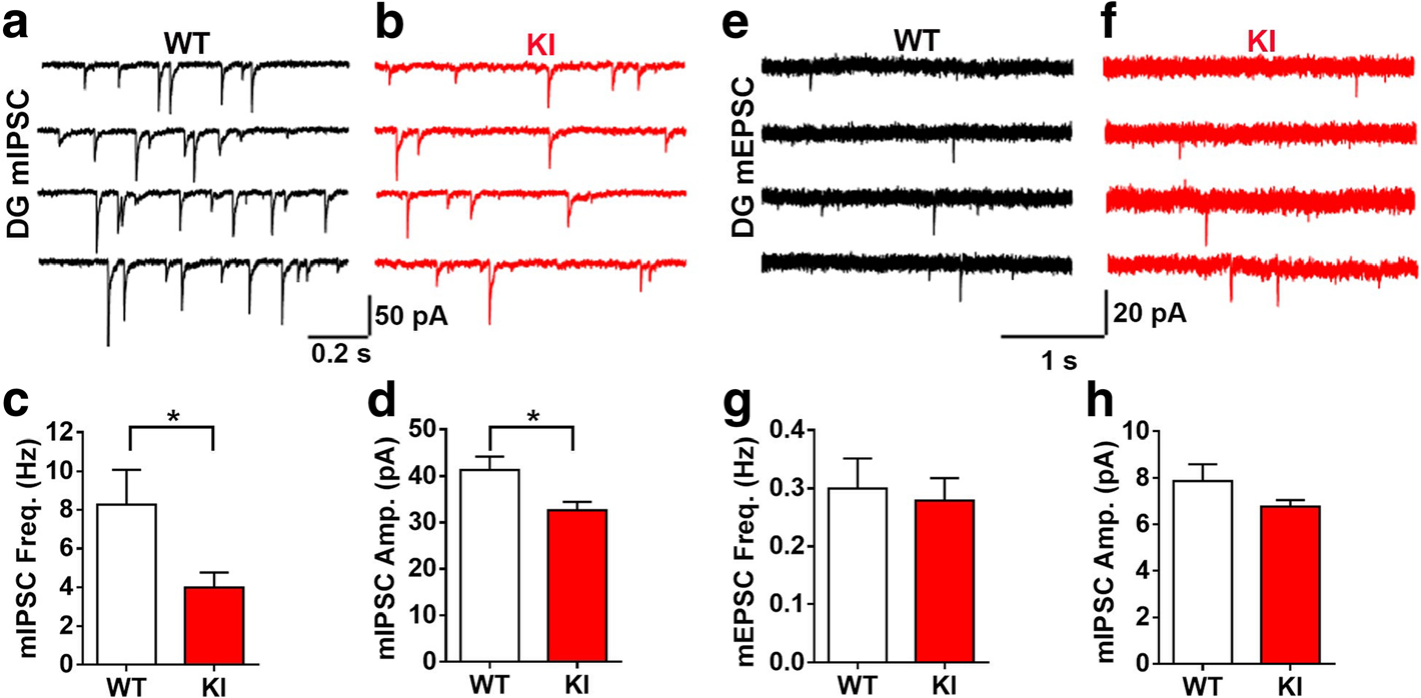
NL2 R215H KI mice have decreased inhibitory synaptic transmission at the hippocampal region. **a**, **b** Representative traces of miniature inhibitory postsynaptic currents (mIPSCs) recorded from DG granule cells in hippocampal slices of WT (black) and R215H KI (red) mice. WT, *n* = 14 cells / 4 mice; R215H KI, *n* = 18 cells / 4 mice. **c**, **d** Quantification of the mIPSC frequency and amplitude (Student’s *t*-test). **e**, **f** Representative traces of miniature excitatory postsynaptic currents (mEPSCs) in the DG region of hippocampal slices from WT (black) and R215H KI (red) mice. WT, *n* = 11 cells / 4 mice; KI, *n* = 12 cells / 3 mice. **g**, **h** Quantification of the mEPSC frequency and amplitude (Student’s *t*-test). Data represent mean ± SEM; *P < 0.05, **P < 0.01, ***P < 0.001

We next investigated the function of inhibitory neurotransmission in the R215H KI mice. Whole-cell patch-clamp recordings were performed on dentate granule cells in acute brain slices of adult WT and homozygous R215H KI mice. We found that both the frequency and amplitude of miniature inhibitory postsynaptic currents (mIPSCs) were significantly decreased in the granule cells of R215H KI mice (Fig. 4a-d; Frequency: WT = 8.28 ± 2.21 Hz, KI = 3.98 ± 0.78 Hz, *p* = 0.041; Median amplitude: WT = 41.3 ± 2.9 pA, KI = 32.7 ± 1.8 pA, *p* = 0.019; Student’s *t*-test). In contrast, there was no significant change of miniature excitatory postsynaptic currents (mEPSCs) in the dentate granule cells of R215H KI mice compared to WT mice (Fig. 4e-h). The kinetics of both mIPSCs and mEPSCs were not altered (Additional file 1: Figure S7). These Results indicate that NL2 R215H mutation is primarily affecting inhibitory neurotransmission.

### Behavioral deficits in NL2 R215H KI mice

**Fig. 5:**
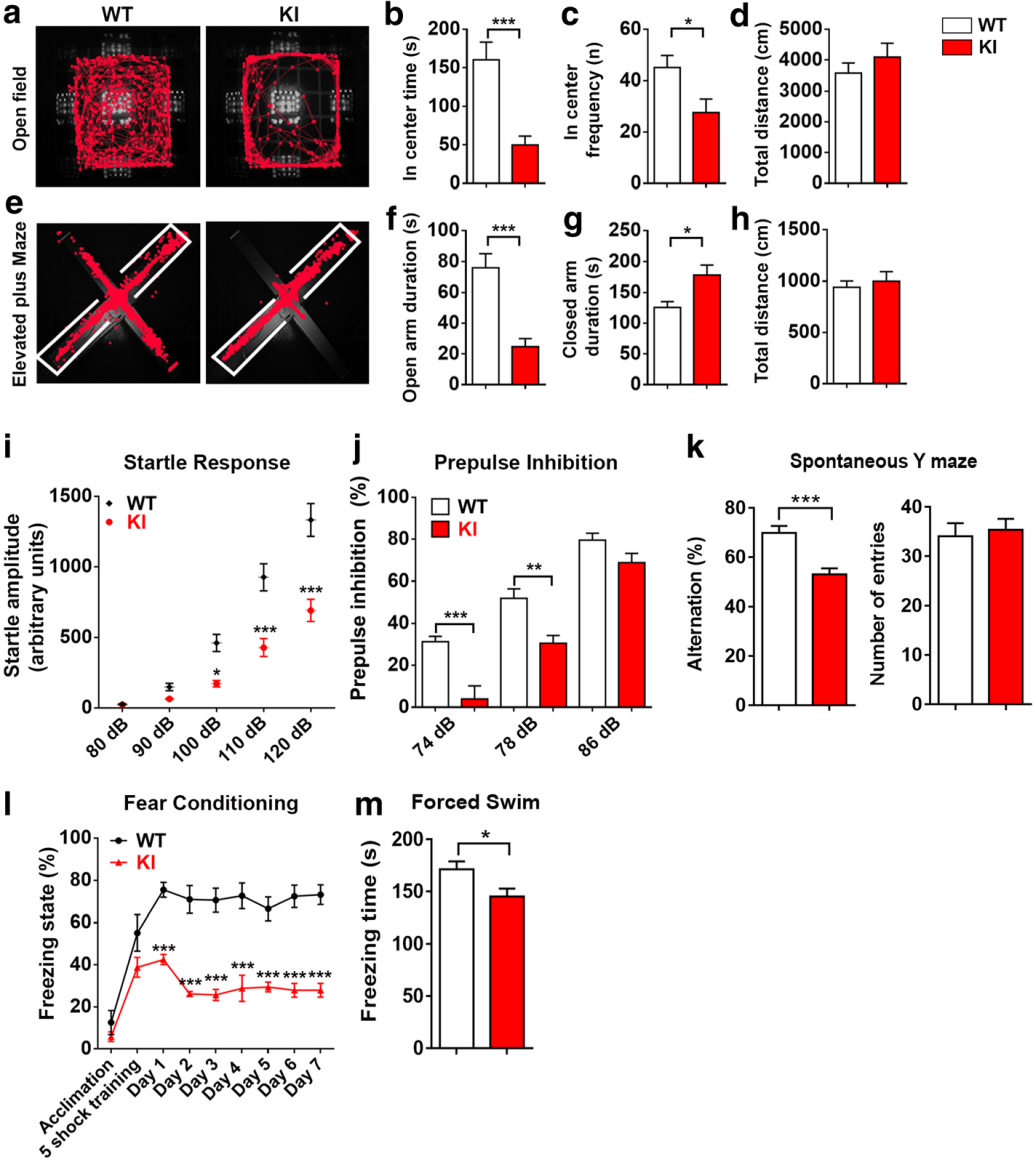
NL2 R215H KI mice display schizophrenia-like behaviors. **a** Representative running track of WT and R215H KI mice (male) in an open field within 10 min duration. **b** The center time of WT and KI mice spent in the open field. **c** The frequency of WT and KI mice entering the center zone of the open field. **d** The total distance of WT and KI mice traveled in the open field test. **e** Representative running track of WT and R215H KI mice (male) in elevated plus maze for 5 min. White line indicates closed-arms. **f** The quantified time spent in the open-arms of WT and KI mice. **g** The time spent in the closed-arms of WT and KI mice. (**h**) The total distance traveled in the elevated plus maze test. **a**-**h** WT mice *n* = 11, KI mice *n* = 12,; Student’s *t*-test was used for analysis. **i** Startle response of WT and R215H KI mice (male) toward 80, 90, 100, 110, and 120 dB sound pulses. **j** The percentage of pre-pulse inhibition (PPI) to a pre-pulse of 74 dB, 78 dB, and 86 dB. WT mice *n* = 12, KI mice *n* = 9. Two-way ANOVA with Sidak’s multiple comparison test was used for analysis. **k** Spontaneous Y maze test. WT mice *n* = 10, KI mice *n* = 12, Student’s *t*-test. **l** Contextual fear conditioning test. R215H KI mice exhibit significant reduction of freezing time when placed back in the test chamber after 1–7 days of shock training (Two-way ANOVA with Sidak’s multiple comparison test, genotype F_(1, 99)_ = 172.7, *P* < 0.0001, WT *n* = 8, KI *n* = 5). **m** Forced swim test. Freezing time were analyzed. WT mice *n* = 23, KI mice *n* = 16, Student’s *t*-test. Data represent mean ± SEM; *P < 0.05, **P < 0.01, ***P < 0.001

The significant reduction of inhibitory neurotransmission in the NL2 R215H mutant mice prompted us to further investigate whether such severe GABAergic deficits will result in any behavioral deficits. We first performed open field test (10 min). We found that the KI mice spent significantly less time in the center region, although the total distance traveled was similar to the WT mice (Fig. 5a-d). Consistently, in the elevated plus maze test, the KI mice spent much less time in the open arm compared to the WT mice, while the total travel distance was also similar between the KI and WT mice (Fig. 5e-h). These results suggest that the R215H KI mice display an increased level of anxiety while their locomotion activity is relatively normal.

We next examined in R215H KI mice the acoustic startle response and pre-pulse inhibition, a standard test for the sensory motor gating function often assessed in schizophrenia patients [5]. R215H KI mice showed a significant reduction in the startle response when stimulated at 100–120 dB (Fig. 5i). Furthermore, the pre-pulse inhibition was significantly impaired in the KI mice compared to the WT mice (Fig. 5j). Together, these deficits of R215H KI mice suggest that this new transgenic mouse model may recapitulate symptoms of schizophrenia patients.

To further characterize the R215H KI mice, we investigated their cognitive functions by spontaneous Y maze and contextual fear conditioning test. In the Y maze test, we found that R215H KI mice displayed a significant reduction of spontaneous alternation compared to the WT mice (Fig. 5k), indicating a working memory dysfunction. In the contextual fear conditioning test, while the KI mice were capable to associate the conditioning chamber with foot-shock in the initial training, indicated by an increase of freezing state after foot-shock, they failed to retain the fear context memory in the following days when tested (Fig. 5l), indicating an impaired hippocampal dependent cognitive function. Furthermore, we performed forced swim test to investigate whether R215H KI mice have any depression-like behavior, because certain SCZ patients display depression symptom. Interestingly, we observed a reduction of freezing time in KI mice when performing the forced swim test (Fig. 5m), consistent with our observation below that the KI mice show hyperactivity induced by acute stress. The behavioral data shown above was all obtained from male mice, and the female mice were also tested and exhibited the same trend (Additional file 1: Figure S8).

### Impaired hippocampal activation toward acute stress in NL2 R215H KI mice

**Fig. 6:**
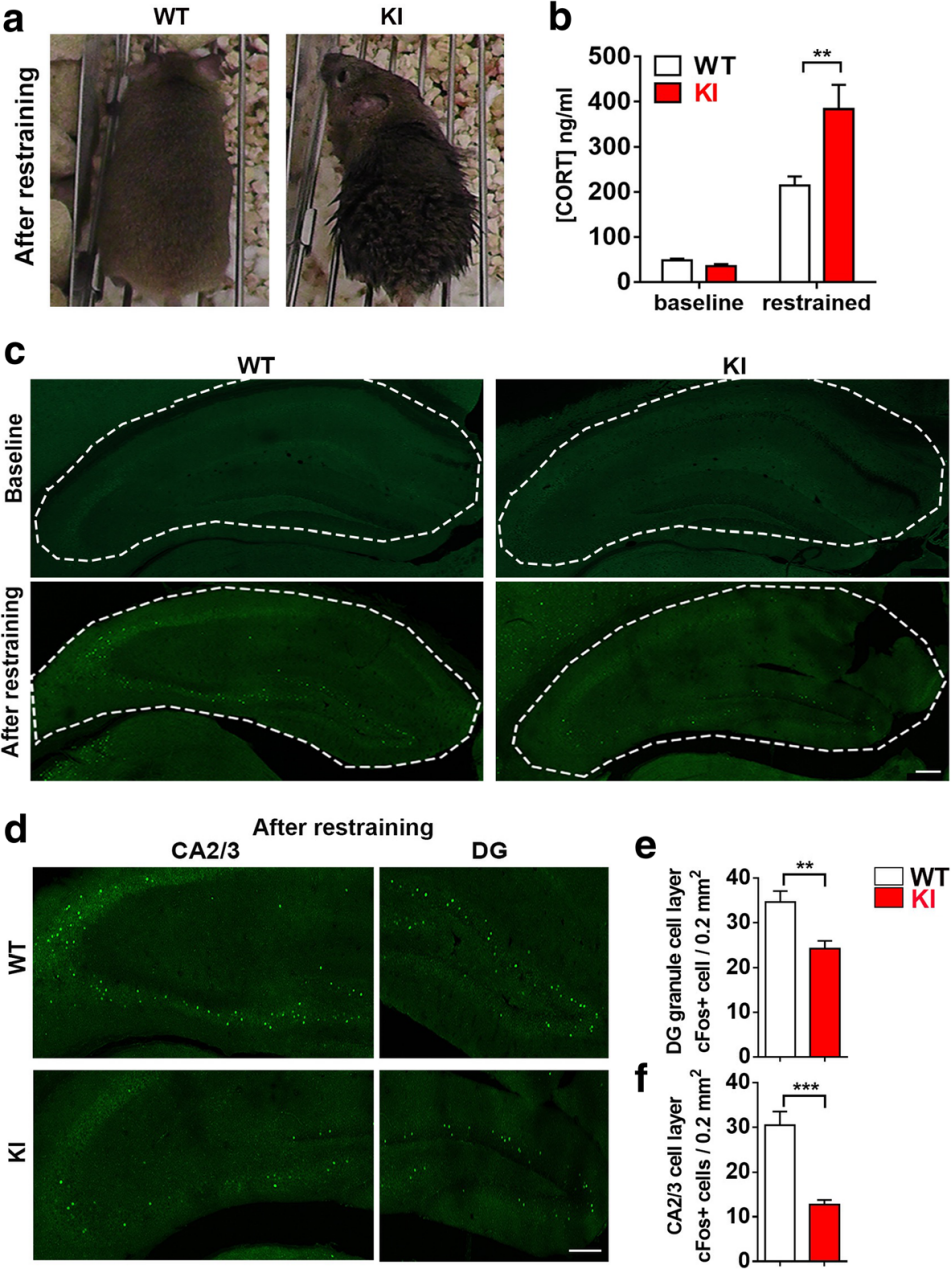
Hippocampal neurons have impaired activation toward acute stress in NL2 R215H KI mice. **a** Typical appearance of WT and KI mice after restraining. **b** Quantified corticosteroid level of WT and KI mice at the baseline level and after 1 h restraining. 4 to 8 mice were used for each genotype at each condition, age 4 to 6 months. Two-way ANOVA with Sidak’s multiple comparison test was used for analysis. **c** Upper row: representative images of baseline cFos immunoreactivity of WT and R215H KI hippocampus; bottom row: cFos immunoreactivity of WT and R215H KI hippocampus after restraining. **d** enlarged DG and CA2/3 region of WT and R215H mice after restraining. **e** Quantification of cFos-positive cells at DG granule cell layers. **f** Quantification of cFos positive cells at CA2/3 pyramidal cell layers. WT: *n* = 16 brain slices / 4 mice; R215H KI: *n* = 20 brain slices / 5 mice; scale bar = 200 μm. Student’s *t*-test was used for analysis. Data represent mean ± SEM; *P < 0.05, **P < 0.01, ***P < 0.001

Schizophrenia is associated with abnormal response to stress [60]. Stress is known to activate the hypothalamic-pituitary-adrenal axis (HPA axis) and induce the hormone release of corticosterone (CORT) into circulation [34, 41]. To investigate the stress response of R215H KI mice, we put the WT and R215H KI mice into restraining tubes for 1 hour as an acute stress test. We found that R215H KI mice struggled much more intensively for a long time and excreted much more than the WT mice during the restraining test. After restraining, KI mice were more dirty and stinky than the WT mice (Fig. 6a). In accordance, R215H KI mice showed a much higher level of CORT (384 ± 53 ng/ml) after restraining compared to the WT mice (215 ± 20 ng/ml). The baseline level of CORT was similar between WT (49 ± 4 ng/ml) and KI mice (36 ± 4 ng/ml) (Fig. 6b; *p* = 0.0035 after restraint, Two way ANOVA followed with Sidak’s post hoc test). These results suggest that R215H KI mice have hyperactive HPA response toward stress.

Following the activation of HPA axis, hippocampus will be activated as a negative feedback regulator and control the CORT level within normal range [23, 58]. To examine the hippocampal activation in R215H KI mice following the acute stress, we used a naïve cohort of mice to perform the restraining test again. R215H KI and WT mice were subjected to restraint for half an hour and then sacrificed after 2 h. Hippocampal activation was examined by assessing the expression level of an immediate early gene cFos [42, 48]. At the baseline level, very few cFos-positive neurons were detected in the hippocampal regions in both WT and KI mice (Fig. 6c, top row). After stress, we observed a significant increase of cFos-positive cells in the DG and CA2/3 regions of the hippocampus in WT mice (Fig. 6c, bottom left). In contrast, the R215H KI mice showed much reduced cFos-positive cells in the same regions of hippocampus (Fig. 6c, bottom right). This is better illustrated in the enlarged images showing the CA2/3 and DG regions of WT mice (Fig. 6d, top row) and KI mice (Fig. 6d, bottom row). Quantitative analysis confirmed the reduction of cFos-positive cells in both CA2/3 (Fig. 6e) and DG (Fig. 6f) regions in the KI mice. These results suggest that NL2 R215H KI mice had impaired hippocampal activation during acute stress.

## Discussion

In the present study, we generated a unique mouse model carrying a single point mutation R215H of *NLGN2* gene that was originally identified from human schizophrenia patients. The NL2 R215H KI mice have impaired GABAergic synapse development, reduced inhibitory synaptic transmission, and decreased hippocampal activation in response to stress. Moreover, the R215H KI mice display anxiety-like behavior, impaired pre-pulse inhibition, cognitive deficits and abnormal stress response, partially recapitulating some of the core symptoms of schizophrenia patients. These results suggest that this newly generated R215H KI mouse line may provide a unique animal model for studying molecular mechanisms underlying schizophrenia and related neuropsychiatric disorders.

### GABAergic and behavioral deficits in NL2 R215H KI mice

NL2 plays important roles in regulating perisomatic GABAergic synapse development, phasic GABAergic transmission, and neural excitability [1, 3, 9, 19, 24, 25, 30, 40, 47, 59, 61]. Consistent with our previous in vitro studies, the current in vivo work demonstrates that R215H mutation disrupts GABAergic synapse development. Functionally, NL2 R215H mutation caused a reduction of both frequency and amplitude of inhibitory neurotransmission. These results suggest that the R215H KI mice display more GABAergic deficits than the reported NL2 KO mice [1, 9, 19, 30, 47], which might explain why our KI mice display more behavioral deficits than the NL2 KO mice, such as PPI impairment, cognitive deficits, and abnormal stress response. Coincidentally, previous studies reported that NL3 R451C KI mouse also displayed stronger phenotypes than the NL3 KO mice [14, 16, 57, 64]. These evidences suggest that genetic mouse models based on mutations identified from patients may be more suitable than the germline KO mouse models for studying pathological mechanisms of human diseases, because of less compensation from other genes in KI mice than in KO mice.

Behaviorally, NL2 R215H KI mice display an anxiety phenotype, which may be the result of decreased GABAergic inhibition [3, 11, 63]. Interestingly, R215H KI mice also show impaired startle responses and deficits in pre-pulse inhibition (PPI). Previous study in rats has reported that disturbance of PV neuron development in the hippocampal DG region may cause reduction of PPI [21]. A recent study also demonstrates that specific inhibition of PV neurons in the ventral hippocampus results in a reduction of both startle response and PPI [45]. Consistent with these findings, we demonstrate here that our R215H KI mice display a significant reduction of PV innervation in the hippocampus, which may underlie the deficits of PPI. In contrast, the NL2 KO mice lack PPI deficit, which might be related to an insufficient loss of PV innervation at hippocampal regions [61]. Besides PV neurons, CCK (cholecystokinin) neurons are another type of inhibitory neurons mainly innervate CA1/2/3 pyramidal cells and DG proximal dendrites. CCK neurons can release GABA to act on GABA_A_ receptor α2 subunits that are known to mediate anxiolytic effect, or release CCK to act on CCK2 receptors and induce anxiogenic effect [18]. It would be important to further investigate the expression and functional alteration of CCK neurons in our R215H KI mice in future studies.

Surprisingly, our former collaborator Dr. Chia-Hsiang Chen’s group recently reported that their NL2 R215H KI mice displayed an increased pre-pulse inhibition phenotype [8]. However, because the startle response of their KI mice was not reported, it makes the data difficult to compare with ours. Additionally, they reported that their KI mice didn’t express NL2 and resembled global NL2 KO mice, but they did not present the actual comparison with NL2 KO mice. In contrast, our R215H KI mice are clearly different from the NL2 KO mice, because our KI mice showed small amount of NL2 expression, particularly during early developmental stages. The low expression level of NL2 in our R215H KI mice distinguishes our KI mice from the NL2 KO mice, which showed completely absent expression of NL2 in our Western blot analysis. Furthermore, our R215H KI mice also showed clear GABAergic deficits as expected, but it is unknown whether their KI mice have any GABAergic deficits or not [8].

Another interesting observation is that the R215H KI mice are hyperactive after acute stress and are associated with impaired hippocampal activation. It has been reported that robust neuron activation requires low background activity before stimulus [33, 49]. However, due to the reduction of GABAergic inhibition in our R215H KI mice, the background activity of hippocampal neurons may be chronically elevated, which will dampen further activation of the hippocampus by external stimulation [40]. The impaired activation of hippocampal neurons in R215H KI mice may contribute to the abnormal stress response we observed, as hippocampus acts like a “brake” during acute stress to prevent HPA axis from over activation [22]. Besides hippocampus, sensitized HPA-axis involves brain regions such as hypothalamus and amygdala. Loss of NL2 in these areas could directly affect their GABAergic transmission and releasing of corticosteroids into the circulation, which is worth of further investigation as well.

### NL2 R215H mutation and schizophrenia

It is well documented that schizophrenia patient’s show impaired pre-pulse inhibition as an abnormal sensorimotor gating deficit [5, 20]. Many patients also have emotional symptoms such as anxiety and depression [39]. Additionally, patients are hypersensitive toward stress and certain patients have been found with altered HPA axis function [4]. Intriguingly, R215H KI mice recapitulated these SCZ-like behaviors, suggesting a potential role of NL2 R215H in the development of schizophrenia symptoms. Furthermore, reduction of PV expression and PV-positive synapses is a prominent phenotype observed in SCZ patients [36–38, 62]. The R215H mutation KI mice also show a significant reduction of PV innervation, consistent with the pathogenic deficit of SCZ patients. These GABAergic deficits, together with cognition and PPI deficits manifested in the KI mice, support the hypothesis that GABA dysfunction makes an important contribution to the cognitive and attention deficits of SCZ. Taken together, NLGN2 R215H single point mutation has a significant impact on GABAergic synapse development and the pathogenesis of neuropsychiatric disorders. Our newly generated NL2 R215H KI mice may provide a useful mouse model for the study of molecular mechanisms and drug development of neuropsychiatric disorders including schizophrenia.

## Methods

### NL2 R215H knock-in mice

The NL2 R215H knock-in mice were generated by homologous recombination in embryonic stem cells by Dr. Siu-Pok Yee’s team at the University of Connecticut Health Center. NL2 KO mice were purchased from Jackson Laboratory (stock# 008139). The detailed procedures are described in the Additional file 1.

All the experimental mice were group housed (2–3 mice per cage) in home cages and lived at a constant 25 °C in a 12 h light/dark cycle. Mice were given ad libitum access to food and water. Littermate or age and gender matched mice were used for experiments. All animal care and experiments followed the Penn State University IACUC protocol and NIH guidelines.

### Biochemical measurements

Protein levels were quantified using total brain homogenates from 3 groups of adult male littermates- WT, heterozygous and homozygous. The western blot system used was the standard Bio-Rad mini protein electrophoresis system and the procedure followed the system manual. LiCOR Odyssey Clx was used for protein signal detection. The antibodies used were Rb anti-Neuroligin 2 (1:1000, SYSY 129202), Rb anti-GAPDH (1:10000, Sigma G9545), and Gt anti-Rb 800 (1:15000, P/N 925-32210, P/N 925-32211). Detailed procedures are described in the Additional file 1.

### Immunohistochemistry, image acquisition, and image analysis

Mouse brain slices were prepared at 20–40 μM and reacted with the primary antibodies Rb anti-Neuroligin 2 (1:1000, SYSY129203), Ms. anti-Parvalbumin (1:1000, MAB1572), GP anti-vGAT (1:1000, SYSY 131004), Gephyrin (1:1000, SYSY 147011), GABAaR γ2 (1:1000 SYSY 224003), and c-Fos (1:5000 Sigma F7799). The fluorescent secondary antibodies used were Gt anti-Rb 488, Gt anti-Ms Cy3, and Gt anti-GP 647. Images were taken with the Olympus FV1000 confocal microscope. The number of neurons and the density and size of synaptic puncta were analyzed with the NIH ImageJ software (NIH, Bethesda, MD, USA). A detailed description of the experimental procedures is in the Additional file 1.

### Slice electrophysiology

Horizontal acute hippocampal slices were used for whole-cell patch clamp recordings. Miniature inhibitory or excitatory postsynaptic currents (mIPSCs or mEPSCs) were pharmacologically isolated by including DNQX and APV or picrotoxin together with tetrodotoxin in artificial cerebrospinal fluid. Details are in the Additional file 1.

### Behavioral tests

#### Overview

The mice for behavior tests were group housed by genotype. All tests were performed during 1 pm to 6 pm. Four cohorts of mice were used: First cohort of mice was first tested for open field, elevated plus maze, and Y maze at 2–3 months old, and then tested for the startle response and pre-pulse inhibition at 3.5 months old. Second cohort of mice was used for contextual fear conditioning test at 2–3 months old. Third cohort of mice was used for restraining and corticosteroid serum level test at 4–6 months old. Fourth cohort of mice was tested for forced swim at 3 months old. The open field test and elevated maze data were analyzed by Noldus Ethovision XT 8.0 software. Y maze and forced swim tests were analyzed with the researcher blind to genotype. Detailed procedures are in Additional file 1*.*

## Additional file

Additional file 1: Supplemental information. (DOCX 6071 kb)

## Abbreviations

APV: D(−)-2-Amino-5-phosphonopentanoic acid
CORT: Corticosterone
DG: Dentate gyrus
DNQX: 6,7-dinitroquinoxaline-2,3-dione
ER: Endoplasmic reticulum
HPA: Hypothalamic-pituitary-adrenal axis
KI: Knock in
KO: Knock out
mEPSC: Miniature excitatory postsynaptic current
mIPSC: Miniature inhibitory postsynaptic current
NL-2: Neuroligin-2
NLGN: Neuroligin
NRXN: Neurexin
PPI: Pre-pulse inhibition
PV: Parvalbumin
SCZ: Schizophrenia
vGAT: Vesicular GABA transporter
WT: Wild type
