# Supplementary material for: GABAergic deficits and schizophrenia-like behaviors in a mouse model carrying patient-derived neuroligin-2 R215H mutation
Source: Mol Brain. 2018 Jun 1;11:31. doi: 10.1186/s13041-018-0375-6 (PMC5984814; doi:10.1186/s13041-018-0375-6)
Supplement: Supplementary file 1 — Supplemental information. (DOCX 6071 kb) [file 13041_2018_375_MOESM1_ESM.docx]

**SUPPLEMENTAL INFORMATION**

**GABAergic deficits and schizophrenia-like behaviors**

**in a mouse model carrying patient-derived neuroligin-2 R215H mutation**

**Dong-Yun Jiang et al.**

**A. DETAILED EXPERIMENTAL PROCEDURES**

**Generation of NL2 R215H KI mice**

A 11.2 kb mouse genomic clone encompassing exons 3 to 8 of neuroligin 2 was retrieved from BAC clone RP24-65N11 and subcloned into pL253 using gap repair method (1). The point mutation G644A (R215H) was introduced into exon 4 by unique restriction site elimination (2). A single LoxP site was inserted into intron 7 approximately 350 bp 3’ downstream of exon 7 by recombineering as described by Liu et al (1), the second LoxP site together with Frt-PGKneo-Frt cassette in reverse orientation was inserted in intron 3 approximately 500 bp 5’ upstream of exon 4. The targeting vector was linearized by NotI digestion and then electroporated into ES cells derived from a F1 (129SvEv X C57BL6/J) embryo. ES cells were then cultured in the presence of G418 and Gancyclovir 48 hours after electroporation. Drug resistant clones were picked into 96-well plates. Genomic DNAs prepared from duplicated plates were used to identify targeted ES clones using nested long-range PCR. The 3’ arm was identified by nested long-range PCR using the primer pair, *Nlgn2* ScR1: 5’- CAACCCCTAGCCTTCCTTACC and 452ScF1: 5’- CTTCTGAGGCGGAAAGAACCA, followed by a second primer pair, *Nlgn2* ScR2: GAGGATGCGGATTCGGCTCCAG and 451ScF2: 5’- CGAAGTTATTAGGTGGATCC, amplifying a 4.56kb fragment. The 5’arm was screened by nested long range PCR using primer pair, Nlgn2 ScF1: 5’- GGTAACTTTACATGCAGGCC and 452ScR1: 5’-TTCCAGACTCCTCATGCCTA followed by a second primer pair, Nlgn2 ScF2: 5’- CCTGTACCTCCTCATTGTGTAC and 452ScR2: GGAATTGGGCTGCAGGAATTCC, amplifying a fragment of 4kb. Three independent targeted ES clones were injected into blastocysts of CD-1 mice to generate chimeric animals and 2 chimeric males derived from each ES clone were used for breeding with ROSA26-Flpe female (Jackson Laboratory stock No. 003946, the flip mice were backcrossed with C57BL6/J over 10 generations) to remove the PGKneo cassette.

Germline transmission was determined by Lox gtF and Lox gtR primer (Lox gtF: 5’-GCAACTTGGTCTGCATCTAAG; Lox gtR: 5’-GAAGGGGAGGGAGAAGTCTGA), This primer pair amplify a fragment of 459 bp specific to mutant allele (in LoxP site) and a fragment of 367 bp specific to wild type allele (in intron 7) (Fig S1a). Genotyping was also confirmed by another primary pair Frt grF (5’-CAGCCATCTAAAGGATTCTTTG) and Frt gtR (5’-GGGCTGGAGGTGCTGGGAGG). The NL-2 R215H mutation was confirmed by sequencing PCR product of I3F primer (5’GTCCCCGATCTCCCGGCCCACC) and E5R primer (5'- GCAGCCTGGTCACCAGTGCTGAG) amplifying the *Nlgn2* exon 4. I3F primer was used for sequencing. PCR product were purified by QIAquick PCR purification Kit (Cat#28104) and sequenced by Penn State Genomics Core facility. The results were viewed and analyzed by sequencing scanner software 1.0 (Fig S1b).

NL2 R215H F1 heterozygotes (offspring of chimera mice X ROSA26-Flpe mice) were mated through non littermate heterozygotes X heterozygotes strategy. The mouse colony was maintained on a mixed genetic background. The wild type, NL2 R215H heterozygotes and NL-2 R215H homozygotes were weaned at postnatal 24 to 28 days.

**Biochemical experiments**

3 groups of male samples were used for analysis. Pierce IP lysis buffer were used for lysing mouse brain tissue (Thermo Scientific Prod# 87788), protease inhibitor cocktail (1: 200 Sigma P8340), phosphatase inhibitor (1: 200 Sigma P5726) and PMSF (1:100 Sigma P7626 10mg/ml) were added in the lysis buffer. Lysates were homogenate on ice using homogenate tube (wheaton 2ml, 7 ml). The sample then were centrifuged at 10,000g 4 ^o^C for 10 minutes. The supernatant was collected and boiled with 4X loading dye (NP0007, life technologies) and 1% of β − Mercaptoethanol at 95 ^o^C for 5 minutes. The sample were directly used for western blot or stored at -20 ^o^C for further use.

**Immunohistochemistry, Image Acquisition and Image Analysis**

Mice were rapidly anesthetized and perfused with ice cold ACSF followed by 40 ml 4% PFA in 0.1M PB, post fixed in 4% PFA overnight in 4 ^o^C. After fixation, brains were dehydrated by 30% sucrose in 0.1M PB. Slices were frozen on sliding microtome at -25 ^o^C and sectioned at 30 um. For c-Fos staining, the mice were restrained for 0.5 hour and waited for 2 hours before perfusion. Brains were post-fixed for 48 hours before dehydration. Slices were sectioned at 40 um.

For gephyrin and GABA_A_ γ2 subunit, the brain slices were prepared following the method described by Schneider Gasser et. al.(3). Briefly, the brain slices were lively sectioned at 300 μm and post fixed for 30 min. After that, the brain slices were rinsed by 1xPBS and dehydrated in 30% sucrose for 3-5 hours. The brain slices were sectioned again at 20 μm in cryostat at -20 ^o^C.

Slices were incubated in blocking solution for 1 hour (0.3% triton 5% NGS in 1X PBS), and then were incubated with primary antibody (dissolved in blocking solution) overnight in 4 ^o^C. On second day, brain slices were washed 3 times with 1X PBS, and then incubated in secondary antibody (dissolved in 0.05% triton 5% NGS in 1X PBS) for 1.5 hours in room temperature. Slices were mounted with mounting solution (Invitrogen P36931). For c-Fos staining, the slices were incubated in 2% triton 10% NGS in PBS for 1 hour followed by primary antibody c-Fos (Sigma F7799) incubation (0.3% triton, 10% NGS in PBS) and secondary antibody incubation (0.05% triton, 10% NGS in PBS).

Synaptic puncta images were acquired by FV 1000 confocal microscope PlAPON 60X O NA 1.42 Objective lens at pixel size of 100 um and optical sections of 1 um. 3 sections were acquired from the subsurface of the brain slice for each image. Images were scanned by sequential line scanning at 8 us/pixel and the image resolution was 1024X1024 pixels with a data depth of 12 bits. Laser transmissivity and channel gain/offset parameters for each antibody were determined by wild type brain slices. All the images were acquired using identical image settings for laser power, channel gain/offset for a given immunostaining marker.

The image was analyzed by ImageJ software. The wild type group of images were analyzed first and a proper threshold were determined for just covering all the synaptic puncta signals. The same threshold was applied to all the images within the same set of immunostaining. Analyze particle function were used for puncta number quantification within granule cell layers or pyramidal cell layers.

**Slice electrophysiology**

For miniature IPSC and EPSC recording at DG region, adult hippocampal slices were prepared from 6 months old male or female littermates following protocol describe by Ting et al. and Zhao et al. (4, 5). NMDG (N-Methyl-D-glucamine) recovery method was slightly modified based on our own experimental condition. Adult mice were quickly anesthetized by 2.5% Avertin and perfused with oxygenated NMDG cutting solution (in mM): 93 NMDG, 93 HCl, 2.5 KCl, 1.2 NaH_2_PO4, 30 NaHCO3, 20 HEPES, 15 Glucose, 5 Sodium ascorbate, 2 Thiourea, 3 Sodium pyruvate, 10 MgSO4.7H_2_O, 0.5 CaCl_2_, 12 N-Acetyl-L-cysteine (PH 7.3-7.4 adjusted by HCl, Osm 300-310), the brains were quickly taken out and cut in the oxygenated NMDG cutting solution at room temperature using Leica 1200S. The 300 um brain slices were recovered at 33.0 ± 0.5 °C in oxygenated NMDG solution for 10-15 minutes. Brain slices were then transferred to the modified HEPES holding aCSF (in mM): 92 NaCl, 2.5 KCl, 1.2 NaH_2_PO4, 30 NaHCO3, 20 HEPES, 15 Glucose, 5 Sodium ascorbate, 2 Thiourea, 3 Sodium pyruvate, 2 MgSO4.7H_2_O, 2 CaCl_2_, 12 N-Acetyl-L-cysteine (PH 7.3-7.4 adjusted by NaOH, Osm 300-310) until recording. Recorded slice was transferred to a submerged recording chamber where they were continuously perfused (3 ml/min) with normal aCSF (in mM: 124 NaCl, 2.5 KCl, 1.2 NaH_2_PO4, 24 NaHCO3, 5HEPES, 13 Glucose, 2 MgSO4.7H_2_O, 2 CaCl_2_, saturated by 95% O2/5% CO2 at 33 °C (TC-324B, Warner instruments Inc). Slices were visualized with infrared optics using an Olympus microscope equipped with DIC optics. For mIPSC recording, the brain slices were incubated in aCSF with 0.5 uM TTX, 50 uM AP5, 10 uM DNQX. The internal pipette solution used was (in mM) 120 CsCl, 5 NaCl, 1 MgCl_2_, 10 HEPES, 0.5 EGTA, 0.3 Na_2_GTP, 3 MgATP. For mEPSC recording, the slices were incubated in ACSF with 0.5 uM TTX and 50 uM picrotoxin. The internal pipette solution was 135 CsMeSO4, 8 NaCl, 0.2 EGTA, 10 HEPES, 4 MgATP, 0.3 Na_2_GTP, 5 Tris-phosphocreatine. The pippette resistance was 3~5 MΩ, access resistance of recorded cells was less than 30 MΩ. Data were collected with a MultiClamp 700A amplifier and pCLAMP9 software (Molecular Devices). The synaptic events were analyzed using Mini Analysis Program (Synaptosoft, New Jersey, USA). over 200 events or 2 minutes recording periods were analyzed for each cell. Events detecting threshold was set at 10 pA. The analysis results were further confirmed visually.

**Behavioral tests**

**Open field test and Elevated plus maze test**

Open field tests were conducted in a 50×50×30 cm^3^ open field arena which was placed in a dark room (with red light). (The camera has built-in infra-red detection). The mice were allowed to roam the enclosure for 10 minutes under video monitoring. The center area was 20×20 cm^2^. The elevated plus maze was 25 cm (open arms) × 25 cm (closed arms) × 30 cm (height) and was placed in the same dark room as the open field test. Dim light (white LED light, 203 mA) was shone upon the maze during the test. The mice were allowed to explore the maze for 5 minutes under video tracking. The number of mice in the cohort: male WT n = 11, male KI n = 12; female WT n = 9, female KI n =10.

**Pre-pulse inhibition test**

The mice were subjected to the startle box once per day for 3 days before the formal test (5-10 minutes per session) in order to adapt them to the experimental environment. For the sound test, 8 trials of pulse at 80 dB (p80 dB), p90 dB, p100 dB, p110 dB, and p120 dB (each pulse for 40 ms) were randomly arranged and given to tested mice with a background noise of 70 dB (SR-Lab San Diego Instruments). The interval time between each trial was randomly set between 10 to 20 seconds by the program. The startle amplitude of the mice toward each pulse was averaged over the 8 trials. Outliers were excluded by Average ± 2*STDEV.

For the pre-pulse inhibition test, the mice first underwent 4 trials of p120. Next, they were subjected to a random ordering of 8 trials of p120 and 8 trials of each pre-pulse p74 dB, p78 dB, p86 dB (each pre-pulse was 20 ms followed by 100 ms of waiting and 40 ms of p120; the startle amplitude was recorded during the p120 window). The interval time between each trial were randomly generated by the program between 10 and 20 seconds. Four trials of p120 dB were given at the end of test. The total program was 25 minutes. The inhibition percentage was calculated by the formula (p120- pp) /p120*100. Outliers were excluded by Average ± 2* STDEV. The number of mice in the cohort: male WT n = 12, male KI n = 9; female WT n = 7, female KI n= 13.

**Spontaneous Y maze test**

Tested mice were placed in a 3 arm Y maze for 5 min, the starting arm were altered for each mouse; Trials in which the mice run through at least 25 total arms were counted for final analysis. The correction rate was calculated as number of correct triads divided by total arms the mice run. One correct triad means visiting 3 different arms consecutively. WT n = 10, KI n = 12.

**Contextual fear conditioning**

Mice were placed in the conditioned chamber (white noise on 55 dB) for 2 minutes before the unconditioned stimulus (foot shock; 2 ms, 0.2 mA) to explore the environment and remember the context. During training, mice were given 5 times of footshock with 1 minute interval. To measure the memory retention of contextual fear conditioning, mice were placed back in the test chamber 1 day after training for 7 days. Male WT mice n = 8, KI mice n = 5; Female WT mice n = 6, KI mice n = 5.

**Forced swim test**

Mice were placed inside a cylinder (height = 40 cm, diameter = 25 cm) filled with water for 5 minutes at room temperature. Freezing time was recorded and analyzed. WT mice n = 23, KI mice n = 16.

**Corticosterone test**

For the baseline corticosterone (CORT) test, experiments were conducted between 3-5 PM (1-3 h before the light turned off), the mice were rapidly anesthetized with isoflurane and decapitated (within 2 min of removing them from their cage). Blood was collected in a BD microcontainer (Cat#365956) and centrifuged for 8 minutes at 8000 rpm. Serum was then collected and stored at −80 ^o^C until use. For the restraining-induced CORT test, mice were anesthetized and decapitated immediately following a 1-hour period of restraint. The blood sample collection procedure was the same as the baseline sample collection. The experiments were conducted within the same time frame as the baseline test. Four to eight mice were used for each genotype at both baseline and restraint conditions.

**SUPPLEMENTARY FIGURES and FIGURE LEGENDS**

**
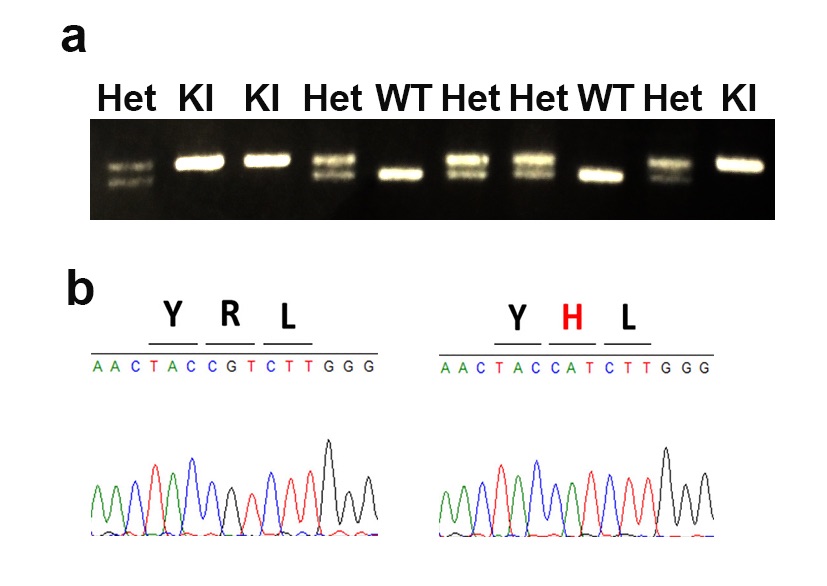
**

**Figure S1** Generation of NL2 R215H KI mice.

(**a**) A representative litter of offspring from R215H Heterozygous mating. Mouse genotype was determined by LoxgtR and LoxgtF primer. (**b**) Identification of NL2 R215H in mouse genome. Left: sequencing of WT *Nlgn2* exon 4 around G644 site. Right: sequencing of NL2 R215H homozygote at the same region as WT, the CGT codon was switched to CAT codon.


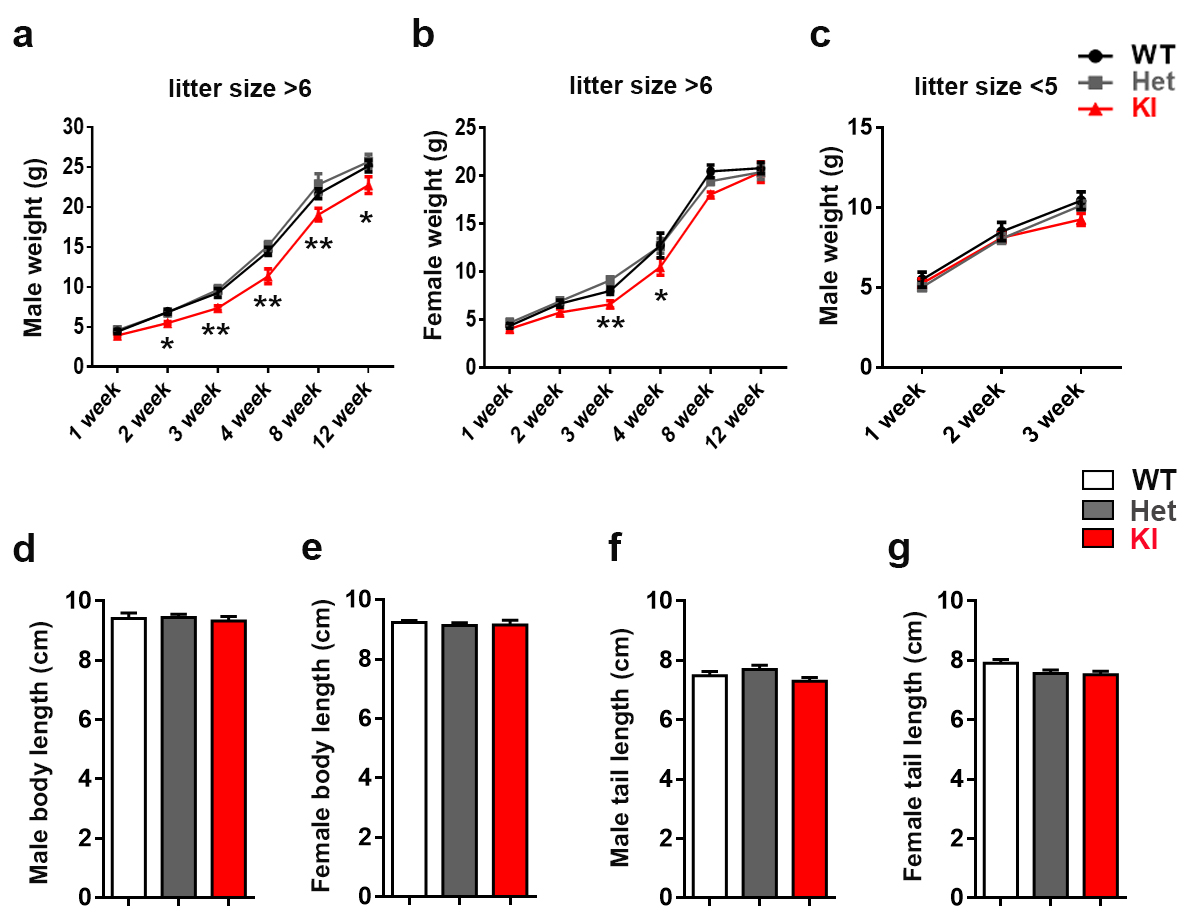


**Figure S2 Developmental time course of body weight in WT, NL2 R215H Het, and NL2 R215H KI mice**.

(**a**) The body weight of male mice in large littermate size (>6) measured from the 1^st^ week to 12^th^ week. WT = 15, Het = 12, KI = 16. (**b**) Body weight of littermate female mice from 1^st^ week to 12^th^ weeks. WT = 12, Het = 8, KI = 10. (**c**) Body weight of littermate male mice grew in littermate size <5: WT = 8, Het = 10, KI n = 7. (**d & f**) Body and tail length of littermate male mice, WT = 11, Het = 9, KI = 10. (**e & g**) Body and tail length of littermate female mice, WT = 11, Het = 15, KI = 12. Two way ANOVA test with Tukey multi-comparison test was used for statistical analysis. Data were shown as Mean ± SEM. *P < 0.05, **P < 0.01.

**
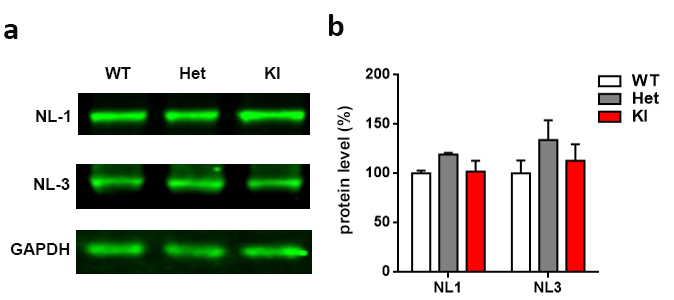
**

**Figure S3 No change of NL1 and NL3 expression in NL2 R215 Het and KI mice.**

(**a**) Representative immunoblots of NL1 and NL3 protein expression in total brain homogenates of WT, NL2 R215H Het, and NL2 R215H KI mice. GAPDH was used as internal control. (**b**) Quantification of NL1 and NL3 expression level in littermate mice. WT n = 4, Het n = 4, KI n = 4. One way ANOVA with Tukey multi-comparison test was used for statistical analysis. Data were shown as Mean ± SEM.


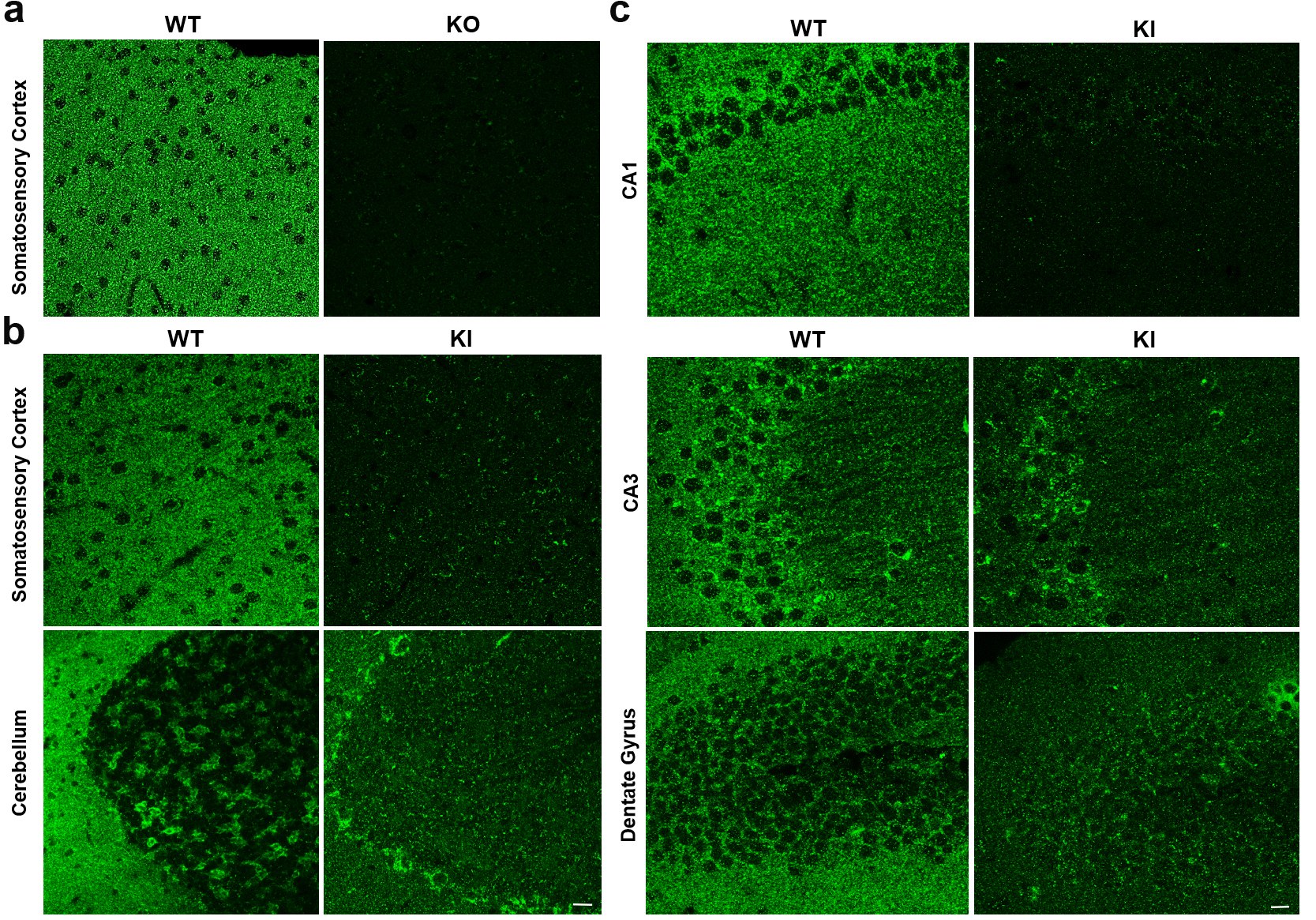


**Figure S4 A significant reduction of NL2 expression level in R215H KI mice.**

(**a**) Representative images of NL2 immunohistochemistry in WT and NL2 global KO mice. (**b-c**) Representative images of NL2 signal in WT and R215H KI mice, shown with different brain regions including somatosensory cortex, cerebellum, and hippocampal regions. Scale bar = 10 μm. The same image acquisition parameters used for NL2 KO brain slices were applied to R215H KI brain slices.

**
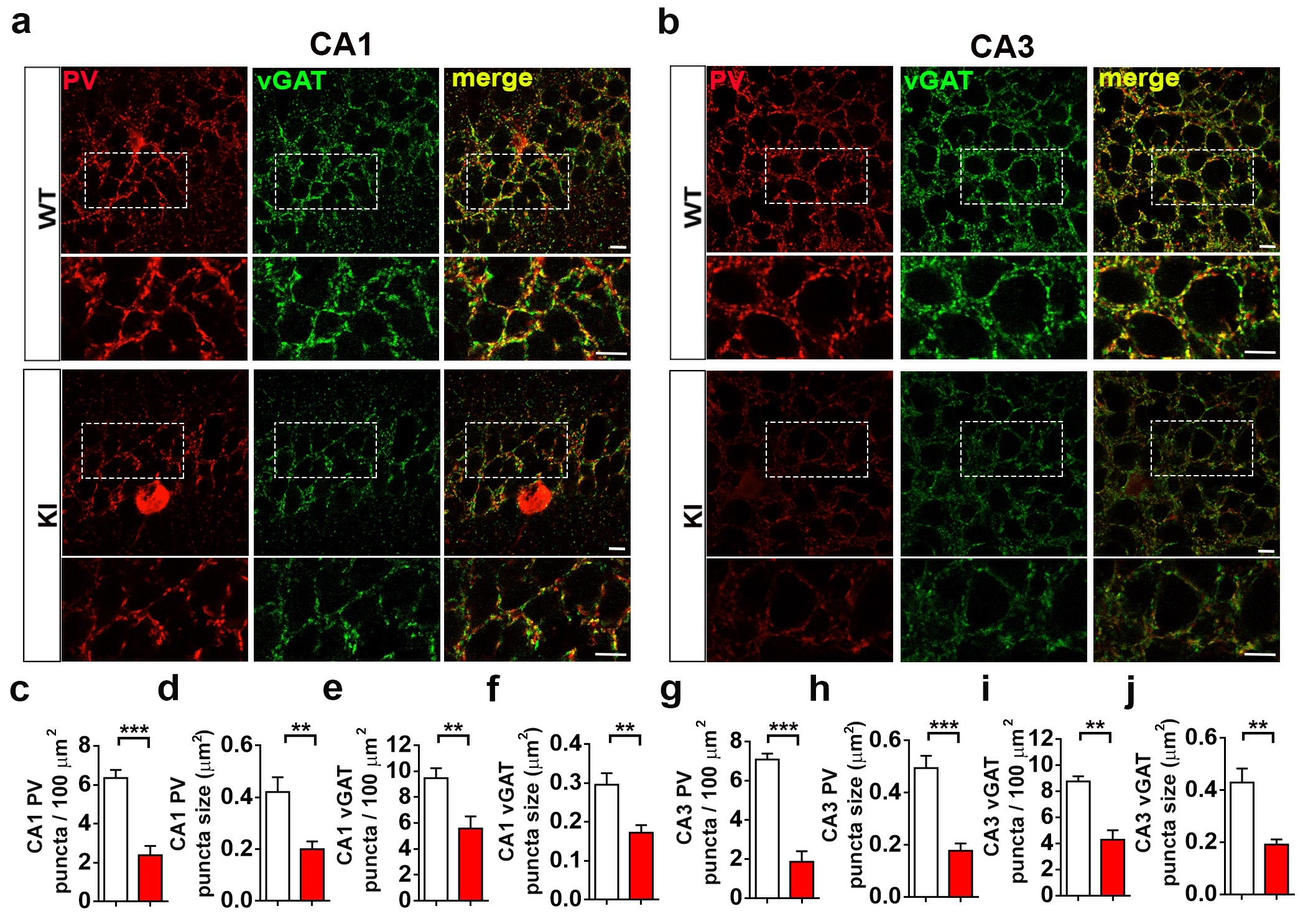
**

**Figure S5 Parvalbumin and vGAT signal significantly reduced in hippocampal CA1/CA3 regions of the NL2 R215H KI mice.**

(**a**) Representative image of PV and vGAT staining at CA1 region in WT and R215H KI mice. Scale bar = 10 um. (**b**) Representative image of PV and vGAT staining at CA3 region in WT and R215H KI mice. Scale bar = 10 um. (**c-f**) CA1 pyramidal cell soma region PV and vGAT puncta quantification. (**g-j**) CA3 cell soma region PV and vGAT puncta quantification. WT n = 9 slices / 3 mice; KI n = 7 slices / 3 mice; Student t test was used for data analysis. Data were shown as Mean ± SEM. **P < 0.01, ***P < 0.001.


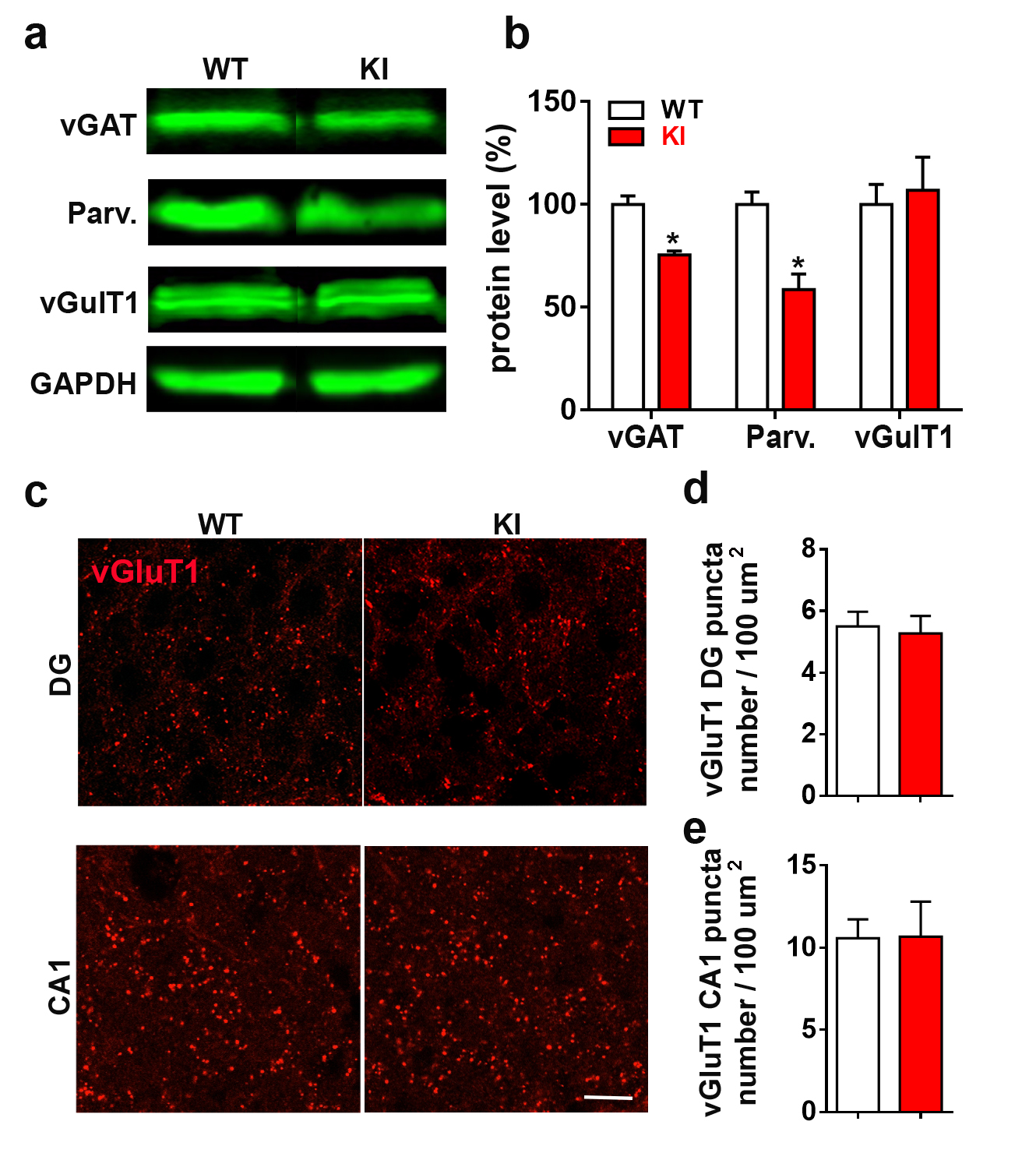


**Figure S6 GABAergic presynaptic marker was reduced in NL2 R215H KI mice.**

(**a**) Representative immunoblots of vGAT, parvalbumin and vGluT1 in hippocampal tissue of WT and NL2 R215H KI mice. GAPDH was used as internal control. (**b**) Quantification of vGAT, parvalbumin and vGluT1 expression level in littermate mice. WT n = 3, KI n = 3. Student’s *t*-test was used for statistical analysis. (**c**) Representative images of vGluT1 synaptic puncta on DG granule cells and CA1 pyramidal cells in WT and R215H KI mice. Scale bar = 10 um. (**d**-**e**) Quantification of the vGluT1 puncta in DG granule cell and CA1 pyramidal cell soma. WT n = 7 slices / 3 mice; KI n = 7 slices / 3 mice; Student t test was used for data analysis. Data were shown as Mean ± SEM. *P < 0.05.

**
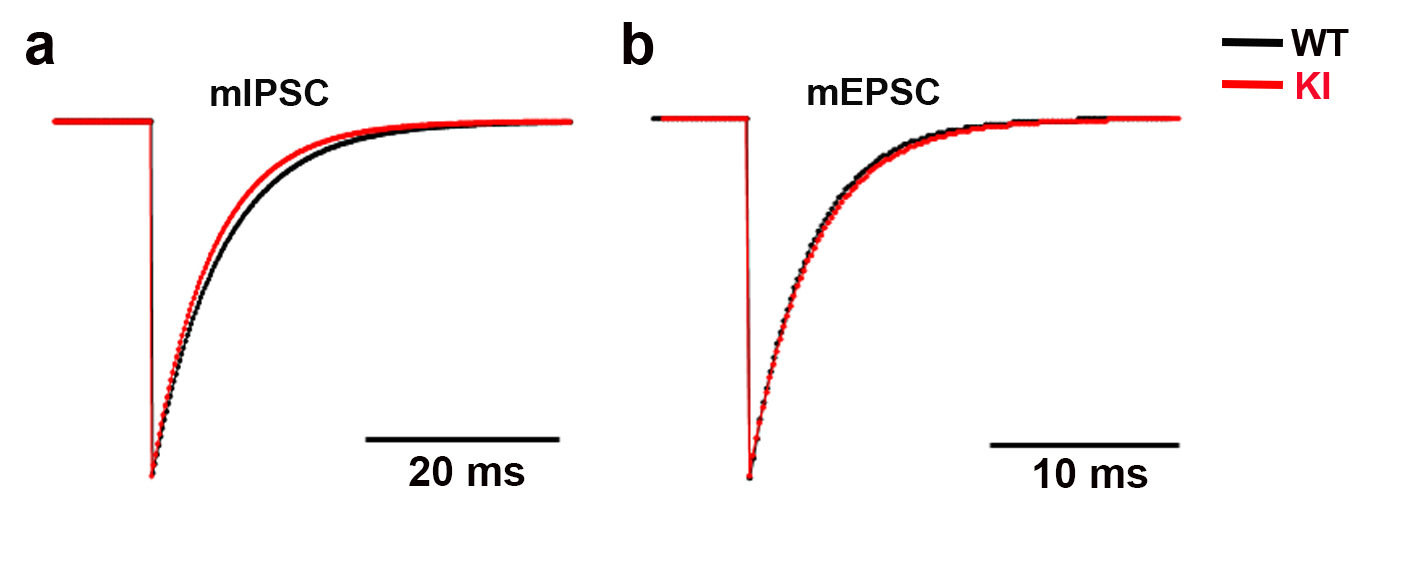
**

**Figure** **S7** **Average traces of mIPSCs and mEPSCs in WT and KI mice.**

(**a**) Overlay of averaged mIPSC traces (scaled) recorded from WT and KI DG granule cells. Fifty events were averaged and aligned for each genotype. (**b**) Overlay of averaged mEPSC traces recorded from WT and KI DG granule cells. Forty events were averaged and aligned for each genotype. Analyzed with Synaptosoft Mini Analysis Program 6.

**
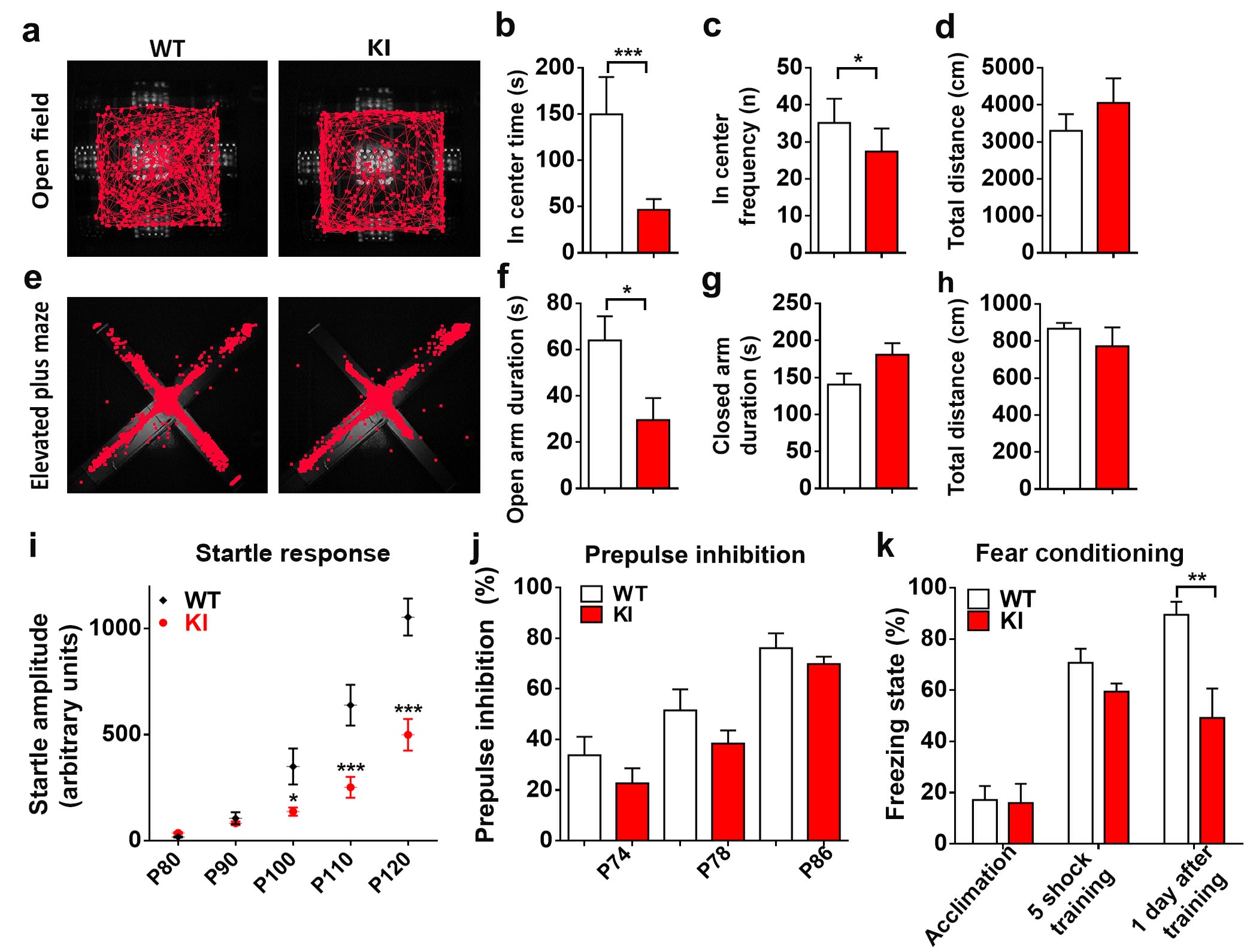
**

**Figure S8 NL2 R215H female mice show schizophrenia-like behaviors.**

(**a**) Representative track of WT and R215H KI female mice running in open field within 10 minutes duration. (**b**) The center time (seconds) of WT and KI female mice spent in open filed within the trial. (**c**) The frequency of WT and KI female mice entering open field center zone. (**d**) The total distance of WT and KI female mice traveled in open field. (**e**) Representative track of WT and R215H KI female mice in elevated plus maze for 5 minutes. (**f**) The quantified time spent in open arms of WT and KI female mice. (**g**) The time spent in closed arms of WT and KI female mice. (**h**) The total distance traveled on elevated plus maze. (**a-d**) WT mice n=9, KI mice n=8; age at 3 months; (**e-h**) WT mice n = 9, KI mice n = 10, age 2 months. Student’s t tests were used for analysis. (**i**) Startle response of WT and R215H KI female mice toward 80, 90, 100, 110, and 120 dB sound pulses. (**j**) The percentage of prepulse inhibition of the startle response to a prepulse of 74 dB, 78 dB, and 86 dB. WT mice n=7, KI mice n=13, age 3.5 months. Two way ANOVA with Sidak’s multiple comparison test was used for analysis. (**k**) R215H KI female mice exhibited significant reduced freezing time 1 day after shock training. Two-way ANOVA with Sidak’s multiple comparison test, All data were shown as Mean ± SEM, *P < 0.05, **P < 0.01, ***P < 0.001

**Supplemental References:**

1. Liu P, Jenkins NA, Copeland NG. A Highly Efficient Recombineering-Based Method for Generating Conditional Knockout Mutations. Genome Research. 2003;13(3):476-84.

2. Noll S, Hampp G, Bausbacher H, Pellegata N, Kranz H. Site-directed mutagenesis of multi-copy-number plasmids: Red/ET recombination and unique restriction site elimination. BioTechniques. 2009;46(7):527-33.

3. Schneider Gasser EM, Straub CJ, Panzanelli P, Weinmann O, Sassoe-Pognetto M, Fritschy JM. Immunofluorescence in brain sections: simultaneous detection of presynaptic and postsynaptic proteins in identified neurons. Nat Protoc. 2006;1(4):1887-97.

4. Zhao S, Ting JT, Atallah HE, Qiu L, Tan J, Gloss B, et al. Cell type-specific channelrhodopsin-2 transgenic mice for optogenetic dissection of neural circuitry function. Nat Methods. 2011;8(9):745-52.

5. Ting JT, Daigle TL, Chen Q, Feng G. Acute brain slice methods for adult and aging animals: application of targeted patch clamp analysis and optogenetics. Methods Mol Biol. 2014;1183:221-42.
